# PD-L1 and CD4 are independent prognostic factors for overall survival in endometrial carcinomas

**DOI:** 10.1186/s12885-020-6545-9

**Published:** 2020-02-17

**Authors:** Shuang Zhang, Takeo Minaguchi, Chenyang Xu, Nan Qi, Hiroya Itagaki, Ayumi Shikama, Nobutaka Tasaka, Azusa Akiyama, Manabu Sakurai, Hiroyuki Ochi, Toyomi Satoh

**Affiliations:** 1 Doctoral Program in Obstetrics and Gynecology, Graduate School of Comprehensive Human Sciences, University of Tsukuba, Tsukuba, Ibaraki, Japan; 2 Department of Obstetrics and Gynecology, Faculty of Medicine, University of Tsukuba, 1-1-1 Tennoudai, Tsukuba, Ibaraki, 305-8575 Japan

**Keywords:** Endometrial carcinoma, PD-1, PD-L1, Survival, Tumor microenvironment

## Abstract

**Background:**

Tumor microenvironment (TME) including the immune checkpoint system impacts prognosis in some types of malignancy. The aim of our study was to investigate the precise prognostic significance of the TME profile in endometrial carcinoma.

**Methods:**

We performed immunohistochemistry of the TME proteins, PD-L1, PD-1, CD4, CD8, CD68, and VEGF in endometrial carcinomas from 221 patients.

**Results:**

High PD-L1 in tumor cells (TCs) was associated with better OS (*p* = 0.004), whereas high PD-L1 in tumor-infiltrating immune cells (TICs) was associated with worse OS (*p* = 0.02). High PD-L1 in TICs correlated with high densities of CD8^+^ TICs and CD68^+^ TICs, as well as microsatellite instability (*p* = 0.00000064, 0.00078, and 0.0056), while high PD-L1 in TCs correlated with longer treatment-free interval (TFI) after primary chemotherapy in recurrent cases (*p* = 0.000043). High density of CD4^+^ TICs correlated with better OS and longer TFI (*p* = 0.0008 and 0.014). Univariate and multivariate analyses of prognostic factors revealed that high PD-L1 in TCs and high density of CD4^+^ TICs were significant and independent for favorable OS (*p* = 0.014 and 0.0025).

**Conclusion:**

The current findings indicate that PD-L1 and CD4^+^ helper T cells may be reasonable targets for improving survival through manipulating chemosensitivity, providing significant implications for combining immunotherapies into the therapeutic strategy for endometrial carcinoma.

## Background

Endometrial cancer is the most common malignancy of female reproductive organs in developed countries, and the incidence is recently increasing [1]. Primary treatment comprises surgery in combination with adjuvant chemotherapy and/or radiotherapy based on the risk stratification for recurrence. The majority of cases are diagnosed at an early stage, and the 5-year survival rate for those with localized disease is 95% [2]. Yet 15–20% of these tumors recur after primary treatment [3]. The 5-year survival rate for those with advanced/recurrent measurable disease is < 10%, and the efficacy of second-line chemotherapy after primary regimens with taxane plus platinum is not more than 15% [4]. Thus, development of novel treatment strategy for those diseases is urgently required.

Programmed cell death-1 (PD-1), immune inhibiting receptor, is expressed on the surface of activated T cells and B cells, and the PD-1 pathway plays critical roles in maintaining immunological self-tolerance [5]. There are two ligands for this receptor, programmed cell death-ligand 1 (PD-L1) and PD-L2. PD-L2 is expressed on activated dendritic cells and macrophages predominantly as well as on tumor cells and B cells, while PD-L1 is expressed on many cell types including immune cells and tumor cells [6]. Tumor cells escape host antitumor immune response through the PD-1/PD-L1 pathway. Recently, therapeutics targeting this immune checkpoint system have shown unprecedented durable clinical responses in various kinds of tumor [7].

A study by Teng et al. on advanced malignant melanomas showed that tumor microenvironment (TME) can be classified based on tumor infiltrating lymphocytes (TILs) and PD-L1 expression: PD-L1^+^ TIL^+^ group of tumors favorably responded to immune checkpoint blockade [8]. Another study on melanomas by Tumeh et al. showed that pre-existing CD8^+^ T cells located at the invasive tumor margin were associated with the expression of PD-1/PD-L1 immune inhibitory system and may predict response to anti-PD-L1 therapy [7]. Regarding ovarian cancer, a study by Webb et al. on high-grade serous ovarian cancer showed that PD-L1 expressed by tumor-associated macrophages (TAM) was significantly associated with favorable disease-specific survival after anti-PD-1 antibody therapy [9]. Darb-Esfahani et al. have shown that PD-1/PD-L1 expressions in high-grade serous ovarian cancer were significantly associated with favorable progression-free survival (PFS) and overall survival (OS) [10]. Another study on ovarian cancer by Hamanishi et al. has shown that high PD-L1 expression on tumor cells and low CD8^+^ T lymphocyte count are independent prognostic factors for poor PFS and OS [11]. Colorectal cancers with microsatellite instability (MSI) were reported to lead to higher mutation burden, with a greater density of CD8^+^ lymphocytes, and to benefit more from pembrolizumab, a kind of anti-PD-1 antibody [12]. Frequency of MSI in endometrial cancer is reportedly 22–33%, higher than cervical (8%) and ovarian (10%) cancers, being highest among gynecologic malignancies [13]. As regards endometrial cancer, the significance of the PD-1/PD-L1 pathway has just begun to be investigated including a number of ongoing clinical trials [14].

There exist varieties of factors in the TME of endometrial carcinoma. The purpose of the current study is to find out the relationships between the TME profile including PD-1/PD-L1 expressions and clinicopathologic features, and to identify predictive biomarkers for the outcome by treatments. Our findings provide significant implications for formulating novel therapeutic strategy for the disease.

## Methods

### Patients and specimens

All patients diagnosed with endometrial carcinoma, who received surgery in the Department of Obstetrics and Gynecology at the University of Tsukuba Hospital between 1999 and 2009, were identified through our database. A total of consecutive 221 patients were included in the present study, and their medical records were retrospectively reviewed. All samples were obtained with opt-out procedure in accordance with the study protocol approved by the Ethics Committee University of Tsukuba Hospital. The study was performed in accordance with the Declaration of Helsinki. A median follow-up duration was 132 months (range, 3–209 months). Follow-up data were retrieved until 2018-7-20. Staging was performed based on the criteria of International Federation of Gynecology and Obstetrics (FIGO, 2008). Endometrioid carcinomas were subclassified into three grades (G1, G2, and G3) according to the FIGO criteria. Treatment of patients was described previously [15]. Table 1 summarizes the patient demographics.

**Table 1:** Patient demographics

Characteristic	Number (*n* = 221)		%
Median age (range)	57 (26–84)		
FIGO stage			
I	144		65
IA		110	50
IB		34	15
II	17		8
III	36		16
IIIA		13	6
IIIC		23	10
IV	24		11
IVA		2	1
IVB		22	10
Histotype			
Endometrioid	196		89
G1		115	52
G2		56	25
G3		25	11
Serous	12		5
Adenosquamous	4		2
Clear cell	4		2
Poorly differentiated	1		0
Undifferentiated	1		0
Mixed epithelial	3		1
Myometrial invasion> 1/2	81		37
Lymphovascular space invasion	84		38
Primary treatment			
Surgery	221		100
Lymphadenectomy		171	77
Lymphnode sampling		21	10
Lymphnode not removed		29	13
Adjuvant chemotherapy	60		27
TC		55	25
CAP		4	2
Adjuvant radiotherapy	58		26

Abbreviations: *FIGO* International Federation of Gynecology and Obstetrics, *TC* paclitaxel and carboplatin combination, *CAP* cyclophosphamide, doxorubicin, and cisplatin combination

### Immunohistochemistry

Immunohistochemical (IHC) procedures were conducted as described previously [15]. Antibodies used are PD-L1 (SP142, rabbit monoclonal, Spring Bioscience, Pleasanton, CA, USA), PD-1 (NAT105, mouse monoclonal, GeneTex, Irvine, CA, USA), CD4 (clone SP35, rabbit monoclonal, Spring Bioscience, Pleasanton, CA, USA), CD8 (clone C8/144B, mouse monoclonal, Nichirei Biosciences, Tokyo, Japan), CD68 (PG-M1, mouse monoclonal, DAKO, Tokyo, Japan), and VEGF (A-20, rabbit polyclonal, Santa Cruz, Dallas, TX, USA). For PD-L1 staining, antigen retrieval was done by autoclaving at 121 °C for 10 min in Tris/EDTA buffer (pH 9.0), and 1st antibody incubation (1:100) was conducted at 4 °C overnight. The corresponding normal endometria or stroma provided an internal positive control, and negative controls without addition of primary antibody showed low background staining.

### IHC scoring

**Fig. 1:**
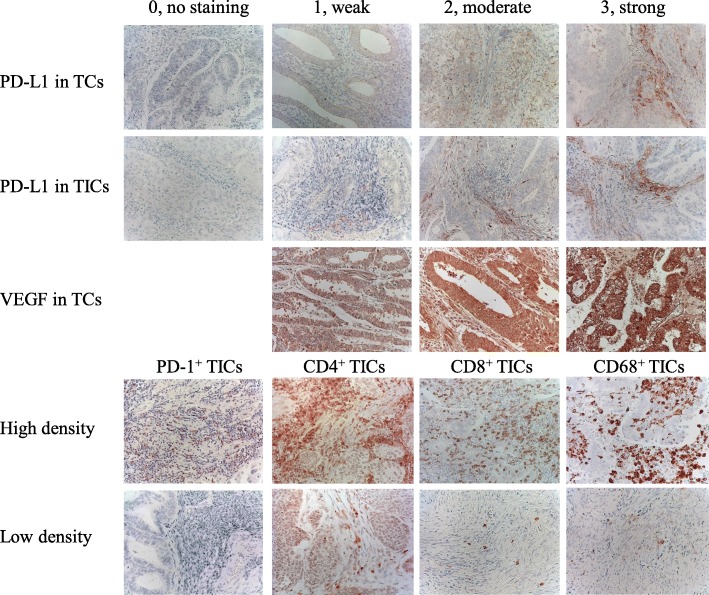
Representative images for immunostaining. The 0 to 3 staining degrees of PD-L1 in TCs/TICs and VEGF in TCs, as well as high and low densities of PD1^+^/CD4^+^/CD8^+^/CD68^+^ TICs. × 200

Blinded for clinical and pathologic parameters, immunoreaction was assigned by two investigators (SZ and TM), and any discrepancies were resolved by conferring over a multiviewer microscope. For semiquantitative analyses for PD-L1 and VEGF, the IHC staining was scored by multiplying the percentages of positive tumor cells (PP: 0, no positive cell; 1, < 10%; 2, 10–50%; and 3, > 50% positive tumor cells) by their prevalent degree of staining (SI: 0, no staining; 1, weak; 2, moderate; and 3, strong). The IHC scores (IHS=PP × SI) range from 0 to 9. For PD-L1, we evaluated membrane staining of tumor cells (TCs) and tumor-infiltrating immune cells (TICs) separately. For CD4, CD8, CD68, and PD-1, we counted positive TICs by magnification of × 200 in most abundant 3 locations of the slide and calculated the average. The representative images for immunostaining are shown in Fig. 1.

### MSI analysis

MSI status was analyzed with the five fluorescence-labeled microsatellite markers, BAT25, BAT26, D2S123, D5S346 and D17S250 [16]. Tumors showing allelic shift at one or more markers were classified as MSI, and tumors with no allelic shift at any marker as microsatellite stable (MSS).

### Statistical analyses

Differences in proportions were evaluated by the Fisher’s exact test. Differences in continuous variables were evaluated by the Mann-Whitney U test. The optimal cut-off values of IHC scores for the relationship with OS were determined by the K-Adaptive partitioning method (Table 2) [17]. Kaplan–Meier survival curves were calculated and compared statistically using the log-rank test. The Cox proportional hazard model was used for the univariate and multivariate analyses. OS was measured from the start of primary treatment to death from any cause. Treatment-free interval (TFI) was measured from the end of primary adjuvant chemotherapy to the diagnosis of recurrence. Statistical analyses were performed using R version 3.5.3.

**Table 2:** Optimal cut-off values of IHC scores for the relationship with OS

	Mean ± SD	Min	Max	Cut-off	Category	N (%)
PD-L1 in TCs	1.36 ± 1.47	0	9	0<	High expression	155 (70)
					Low expression	66 (30)
PD-L1 in TICs	2.70 ± 1.94	0	9	4<	High expression	36 (16)
					Low expression	185 (84)
PD-1^+^ TICs	16.94 ± 23.55	0	129.33	6.67<	High density	131 (59)
					Low density	90 (41)
VEGF in TCs	3.64 ± 1.69	1	9	2<	High expression	151 (68)
					Low expression	70 (32)
CD4^+^ TICs	137.99 ± 65.18	31	391	126.33<	High density	117 (53)
					Low density	104 (47)
CD8^+^ TICs	196.52 ± 121.71	18.33	582.67	296.33<	High density	43 (19)
					Low density	178 (81)
CD68^+^ TICs	161.61 ± 88.00	16.33	527.67	126<	High density	129 (58)
					Low density	92 (42)

Abbreviations: *IHC* immunohistochemical, *OS* overall survival, *SD* standard deviation, *Min* minimum, *Max* maximum, *PD-L1* programmed cell death-ligand 1, *TCs* tumor cells, *TICs* tumor-infiltrating immune cells, *PD-1* programmed cell death-1, *VEGF* vascular endothelial growth factor

## Results

We performed IHC evaluation of the TME proteins in 221 primary endometrial carcinomas (Table 2). First, we examined mutual relationships among the IHC results. High PD-L1 expression in TCs showed an inverse correlation with high PD-L1 expression in TICs (*p* = 0.0054; Table 3). High PD-L1 expression in TICs correlated with high density of PD-1^+^, CD8^+^, and CD68^+^ TICs (*p* = 0.00032, 6.4E-07, and 0.00078; Table 3). High density of PD-1^+^ TICs correlated with high density of CD8^+^, and CD68^+^ TICs (*p* = 0.0097 and 0.00028; Table 3). High density of CD4^+^, CD8^+^, and CD68^+^ TICs showed mutual correlations (Table 3).

**Table 3:** Mutual relationships among TME protein expressions

	PD-L1 in TICs			PD-1^+^ TICs			VEGF in TCs			CD4^+^ TICs			CD8^+^ TICs			CD68^+^ TICs		
	High expression (*n* = 36)	Low expression (n = 185)	*P*-value	High density (*n* = 131)	Low density (*n* = 90)	*P*-value	High expression (*n* = 151)	Low expression (*n* = 70)	*P*-value	High density (*n* = 117)	Low density (*n* = 104)	*P*-value	High density (*n* = 43)	Low density (*n* = 178)	*P*-value	High density (*n* = 129)	Low density (*n* = 92)	*P*-value
High PD-L1 expression in TCs	18 (50%)	137 (74%)	0.0054	88 (67%)	67 (74%)	0.30	102 (68%)	53 (76%)	0.27	86 (74%)	69 (66%)	0.30	33 (77%)	122 (69%)	0.36	88 (68%)	67 (73%)	0.55
High PD-L1 expression in TICs	–	–	–	31 (24%)	5 (6%)	0.00032	29 (19%)	7 (10%)	0.12	24 (21%)	12 (12%)	0.10	19 (44%)	17 (10%)	6.4E-07	30 (23%)	6 (7%)	0.00078
High density of PD-1^+^ TICs	–	–	–	–	–	–	86 (57%)	45 (64%)	0.38	76 (65%)	55 (53%)	0.076	33 (77%)	98 (55%)	0.0097	90 (70%)	41 (45%)	0.00028
High VEGF expression in TCs	–	–	–	–	–	–	–	–	–	76 (65%)	75 (72%)	0.31	33 (77%)	118 (66%)	0.21	88 (68%)	63 (68%)	1.0
High density of CD4^+^ TICs	–	–	–	–	–	–	–	–	–	–	–	–	32 (74%)	85 (48%)	0.0020	77 (60%)	40 (43%)	0.020
High density of CD8^+^ TICs	–	–	–	–	–	–	–	–	–	–	–	–	–	–	–	32 (25%)	11 (12%)	0.024

Abbreviations: *TME* tumor microenvironment, *PD-L1* programmed cell death-ligand 1, *TICs* tumor-infiltrating immune cells, *PD-1* programmed cell death-1, *VEGF* vascular endothelial growth factor, *TCs* tumor cells

Secondly, we examined the relationships between the IHC evaluations and clinicopathologic parameters (Table 4). High PD-L1 expression in TCs was associated with G1, non-G3, superficial myometrial invasion, and negative lymphovascular space invasion (LVI) (*p* = 3.2E-05, 0.00026, 0.0037, and 0.049; Table 4), while high PD-L1 expression in TICs was associated with non-endometrioid histology, non-G1, deep myometrial invasion, positive LVI, and advanced FIGO stage (*p* = 0.0089, 0.018, 0.0044, 0.00026, and 0.014; Table 4). High density of PD-1^+^ TICs was associated with non-endometrioid histology, non-G1, positive LVI, and MSI (*p* = 0.0086, 1.1E-05, 0.0047, and 0.0015; Table 4). High VEGF expression in TCs was associated with deep myometrial invasion, non-stage I, and advanced stage (*p* = 0.00051, 0.0015, and 0.024; Table 4). High density of CD4^+^ TICs was significantly associated with endometrioid histology and superficial myometrial invasion (*p* = 0.033 and 0.00044; Table 4), while high density of CD8^+^ TICs was associated with MSI (*p* = 0.012; Table 4). High density of CD68^+^ TICs showed no significant association with clinicopathologic parameters (Table 4).

**Table 4:** Relationships between TME protein expressions and clinicopathologic parameters

	PD-L1 in TCs			PD-L1 in TICs			PD-1^+^ TICs			VEGF in TCs			CD4^+^ TICs			CD8^+^ TICs			CD68^+^ TICs		
	High expression (*n* = 155)	Low expression (*n* = 66)	*P*-value	High expression (*n* = 36)	Low expression (*n* = 185)	*P*-value	High density (*n* = 131)	Low density (*n* = 90)	*P*-value	High expression (*n* = 151)	Low expression (*n* = 70)	*P*-value	High density (*n* = 117)	Low density (*n* = 104)	*P*-value	High density (*n* = 43)	Low density (*n* = 178)	*P*-value	High density (*n* = 129)	Low density (*n* = 92)	*P*-value
Age > 60	57 (37%)	23 (35%)	0.88	12 (33%)	68 (37%)	0.85	51 (39%)	29 (32%)	0.32	56 (37%)	24 (34%)	0.76	35 (30%)	45 (43%)	0.050	11 (26%)	69 (39%)	0.12	52 (40%)	28 (30%)	0.16
Endometrioid (vs. Non-endometrioid)	141 (91%)	55 (83%)	0.11	27 (75%)	169 (91%)	0.0089	110 (84%)	86 (96%)	0.0086	132 (87%)	64 (91%)	0.50	109 (93%)	87 (84%)	0.033	40 (93%)	156 (88%)	0.43	112 (87%)	84 (91%)	0.39
G1	95 (61%)	20 (30%)	3.2E-05	12 (33%)	103 (56%)	0.018	52 (40%)	63 (70%)	1.1E-05	76 (50%)	39 (56%)	0.47	65 (56%)	50 (48%)	0.28	25 (58%)	90 (51%)	0.40	65 (50%)	50 (54%)	0.59
G3	9 (6%)	16 (24%)	0.00026	7 (19%)	18 (10%)	0.14	17 (13%)	8 (9%)	0.39	21 (14%)	4 (6%)	0.11	12 (10%)	13 (13%)	0.67	7 (16%)	18 (10%)	0.28	17 (13%)	8 (9%)	0.39
MI > 1/2	47 (30%)	34 (52%)	0.0037	21 (58%)	60 (32%)	0.0044	50 (38%)	31 (34%)	0.67	67 (44%)	14 (20%)	0.00051	30 (26%)	51 (49%)	0.00044	13 (30%)	68 (38%)	0.38	53 (41%)	28 (30%)	0.12
LVI	52 (34%)	32 (48%)	0.049	24 (67%)	60 (32%)	0.00026	60 (46%)	24 (27%)	0.0047	64 (42%)	20 (29%)	0.054	46 (39%)	38 (37%)	0.68	18 (42%)	66 (37%)	0.60	55 (43%)	29 (32%)	0.12
FIGO stage I	104 (67%)	40 (61%)	0.36	19 (53%)	125 (68%)	0.125	81 (62%)	63 (70%)	0.25	88 (58%)	56 (80%)	0.0015	81 (69%)	63 (61%)	0.20	32 (74%)	112 (63%)	0.21	82 (64%)	62 (67%)	0.57
FIGO stage III-IV	37 (24%)	23 (35%)	0.10	16 (44%)	44 (24%)	0.014	42 (32%)	18 (20%)	0.064	48 (32%)	12 (17%)	0.024	30 (26%)	30 (29%)	0.65	10 (23%)	50 (28%)	0.57	40 (31%)	20 (22%)	0.17
MSI	29 (19%)	19 (29%)	0.11	12 (33%)	36 (19%)	0.078	38 (29%)	10 (11%)	0.0015	36 (24%)	12 (17%)	0.30	29 (25%)	19 (18%)	0.26	16 (37%)	32 (18%)	0.012	31 (24%)	17 (18%)	0.41

Abbreviations: *TME* tumor microenvironment, *PD-L1* programmed cell death-ligand 1, *TCs* tumor cells, *TICs* tumor-infiltrating immune cells, *PD-1* programmed cell death-1, *VEGF* vascular endothelial growth factor, *MI* myometrial invasion, *LVI* lymphovascular space invasion, *FIGO* International Federation of Gynecology and Obstetrics, *MSI* microsatellite instability

**Fig. 2:**
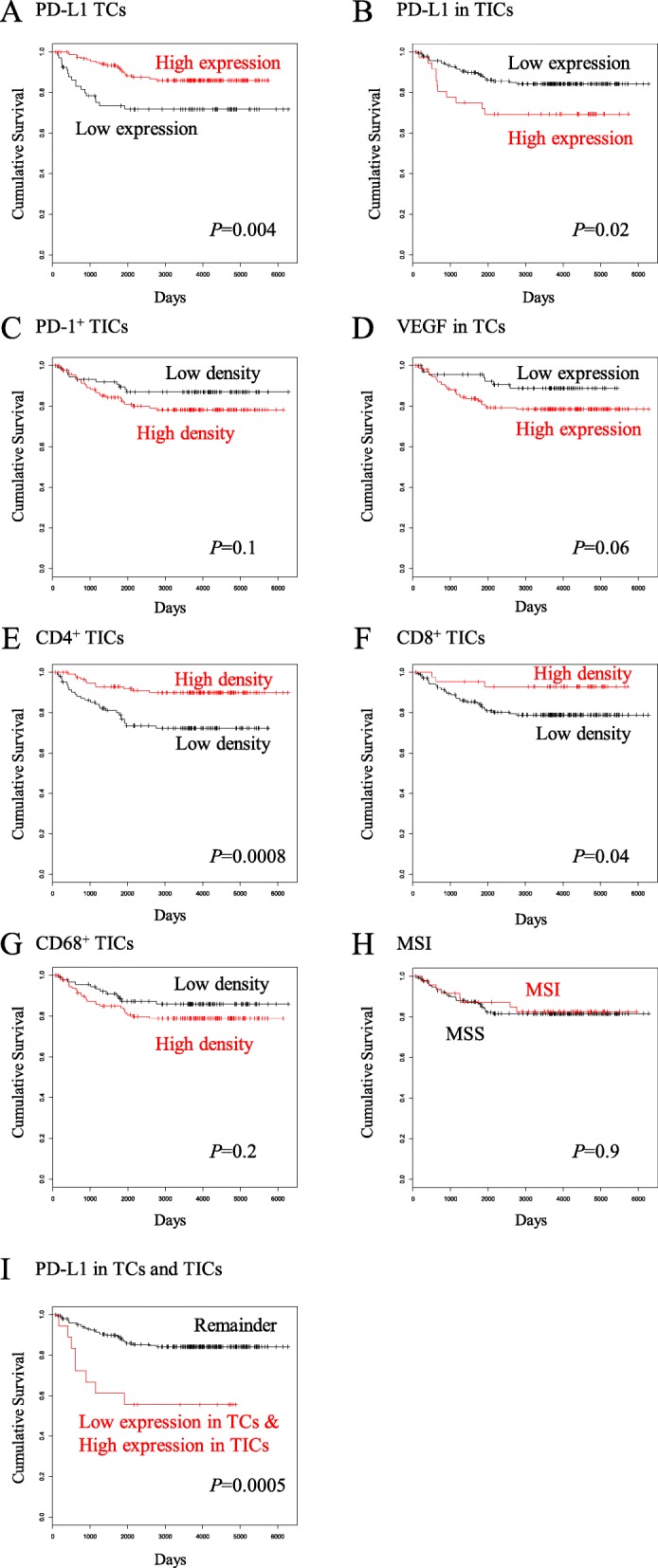
Kaplan-Meier curves for overall survival according to TME protein expressions in endometrial carcinomas. **a**, patients with TCs expressing high PD-L1 (*n* = 74) vs. low PD-L1 (*n* = 147); **b**, patients with TICs expressing high PD-L1 (*n* = 36) vs. low PD-L1 (*n* = 185); **c**, patients with PD-1^+^ TICs of high density (*n* = 81) vs. low density (*n* = 140); **d**, patients with TCs expressing high VEGF (*n* = 151) vs. low VEGF (*n* = 70); **e**, patients with CD4^+^ TICs of high density (*n* = 92) vs. low density (*n* = 129); **f**, patients with CD8^+^ TICs of high density (*n* = 124) vs. low density (*n* = 97); **g**, patients with CD68^+^ TICs of high density (*n* = 105) vs. low density (*n* = 116); **h**, patients with MSI tumor (*n* = 48) vs. MSS tumor (*n* = 173); **i**, patients with TCs expressing low PD-L1 and TICs expressing high PD-L1 (*n* = 18) vs. the remaining patients (*n* = 203)

Thirdly, the patient OS was compared according to the IHC evaluations. Patients with TCs expressing high PD-L1 showed better OS than those with low PD-L1 expression (*p* = 0.004; Fig. 2a), while conversely patients with TICs expressing high PD-L1 showed worse OS than those with low PD-L1 expression (*p* = 0.02; Fig. 2b). High densities of CD4^+^ TICs and CD8^+^ TICs both correlated with better OS (*p* = 0.0008 and 0.04; Fig. 2e and f). As for PD-1^+^ TICs, VEGF in TCs, and CD68^+^ TICs, the OS showed no significant difference (*p* = 0.1, 0.06, and 0.2; Fig. 2c, d, and g). The OS according to MSI/MSS showed no difference (*p* = 0.9; Fig. 2h).

**Fig. 3:**
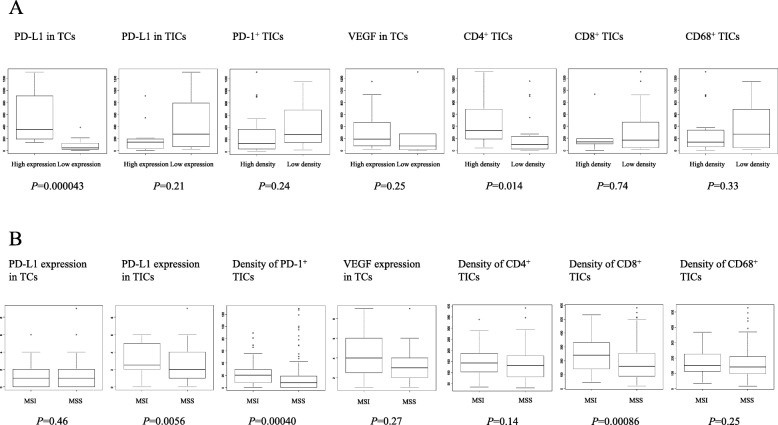
**a**, Comparison of treatment-free interval (days) between patients with TCs expressing high PD-L1 (*n* = 17) vs. low PD-L1 (*n* = 16), those with TICs expressing high PD-L1 (*n* = 13) vs. low PD-L1 (*n* = 20), those with PD-1^+^ TICs of high density (*n* = 24) vs. low density (*n* = 9), those with TCs expressing high VEGF (*n* = 27) vs. low VEGF (*n* = 6), those with CD4^+^ TICs of high density (*n* = 13) vs. low density (*n* = 20), those with CD8^+^ TICs of high density (*n* = 5) vs. low density (*n* = 28), and those with CD68^+^ TICs of high density (*n* = 24) vs. low density (*n* = 9). **b**, Comparison between patients with MSS tumor (*n* = 173) vs. MSI tumor (*n* = 48) of IHC scores of PD-L1 expression in TCs, IHC scores of PD-L1 expression in TICs, density of PD-1^+^ TICs, IHC scores of VEGF expression in TCs, density of CD4^+^ TICs, density of CD8^+^ TICs, and density of CD68^+^ TICs

Next, the associations between TFI after primary adjuvant chemotherapy and the TME protein expressions were examined. High PD-L1 expression in TCs and high density of CD4^+^ TICs were both associated with longer TFI (*p* = 0.000043 and 0.014; Fig. 3a). We further examined the relationships between MSI status and the TME protein expressions. High PD-L1 expression in TICs and high densities of PD-1^+^ TICs and CD8^+^ TICs were associated with MSI (*p* = 0.0056, 0.00040, and 0.00086; Fig. 3b).

Lastly, we conducted univariate and multivariate analyses of prognostic factors for OS. In the univariate analysis, high PD-L1 expression in TICs, older age (> 60), advanced FIGO stage, non-endometrioid histology, deep myometrial invasion (> 1/2), and positive LVI were found to be significant for worse OS (*p* = 0.023, 0.0017, 2.0E-09, 1.9E-07, 6.4E-06, and 0.00011; Table 5), while high PD-L1 expression in TCs and high density of CD4^+^ TICs were significant for better OS (*p* = 0.0050 and 0.0015; Table 5). Subsequent multivariate analysis revealed that high PD-L1 expression in TCs, high density of CD4^+^ TICs, advanced stage, non-endometrioid histology, and positive LVI were significant and independent for OS (*p* = 0.014, 0.0025, 0.000042, 0.0031, and 0.028; Table 5).

**Table 5:** Univariate and multivariate analyses of prognostic factors for OS

	Univariate			Multivariate		
	HR	95% CI	*P*-value	HR	95% CI	*P*-value
High PD-L1 expression in TCs	0.40	0.21–0.76	0.0050	0.43	0.22–0.85	0.014
High PD-L1 expression in TICs	2.25	1.12–4.54	0.023	0.76	0.31–1.82	0.53
High density of PD-1^+^ TICs	1.71	0.85–3.46	0.13	–	–	–
High VEGF expression in TCs	2.14	0.94–4.85	0.07	–	–	–
High density of CD4^+^ TICs	0.32	0.16–0.65	0.0015	0.31	0.15–0.67	0.0025
High density of CD8^+^ TICs	0.31	0.096–1.01	0.053	–	–	–
High density of CD68^+^ TICs	1.58	0.79–3.12	0.19	–	–	–
Age > 60	2.81	1.47–5.36	0.0017	1.36	0.68–2.72	0.39
FIGO stage III/IV (vs. I/II)	8.62	4.26–17.4	2.0E-09	5.50	2.43–12.5	0.000042
Non-endometrioid (vs. Endometrioid)	5.78	2.99–11.2	1.9E-07	3.31	1.50–7.32	0.0031
MI > 1/2	5.04	2.50–10.2	6.4E-06	1.46	0.66–3.19	0.35
LVI (+)	3.75	1.92–7.34	0.00011	2.27	1.09–4.73	0.028

Abbreviations: *OS* overall survival, *HR* hazard ratio, *CI* confidence interval, *PD-L1* programmed cell death-ligand 1, *TCs* tumor cells, *TICs* tumor-infiltrating immune cells, *PD-1* programmed cell death-1, *VEGF* vascular endothelial growth factor, *FIGO* International Federation of Gynecology and Obstetrics, *MI* myometrial invasion, *LVI* lymphovascular space invasion

## Discussion

Our survival analyses exhibited that high PD-L1 expression in TCs was associated with better OS, while conversely high PD-L1 expression in TICs was associated with worse OS (Fig. 2a, b, Table 5). Besides, high PD-L1 expression in TICs showed an inverse correlation with high PD-L1 expression in TCs (Table 3). These findings indicate that PD-L1 expression in TCs and that in TICs seem contrary to each other. PD-L1 expressed on the surface of TCs is supposed to bind to PD-1 receptor on immune cells and to induce adaptive immune resistance. Our above observations may be explicable if some proportion of expressed PD-L1 could move between the surface of TCs and the surface of TICs so that the PD-L1 bound to PD-1 on the surface of TICs may induce adaptive immune resistance leading to poor survival, while the PD-L1 remaining on the surface of TCs may not. This hypothesis may be supported by the published findings that, in addition to tissue PD-L1, there also exist circulating PD-L1 such as exosomal PD-L1 [18, 19] and soluble PD-L1 [20, 21]. However, further molecular and clinical investigations are essential to verify our observation and to elucidate the mechanism underlying them.

High PD-L1 expression in TICs was associated with MSI (Fig. 3b), and with high density of CD8^+^ TICs and CD68^+^ TICs (Table 3), suggesting that PD-L1-induced adaptive immune resistance may involve MSI, killer T cells, and TAMs, as CD8 and CD68 are markers for killer T cells and TAMs, respectively. MSI is known to cause hypermutation leading to increased burden of tumor antigens, which induces increased immune response [13]. Increased immune response may induce PD-1/PD-L1-mediated adaptive immune resistance, which will lead to aggressive tumor phenotype and poor prognosis. Indeed, our analyses of the relationships between the TME protein expressions and clinicopathologic parameters exhibited that high PD-L1 expression in TICs was associated with non-endometrioid histology, non-G1, deep myometrial invasion, positive LVI, and advanced FIGO stage (Table 4), and our survival analysis demonstrated that high PD-L1 expression in TICs was associated with unfavorable OS (Fig. 2b). Taken together, these findings suggest that PD-L1 expression of TICs may be a biomarker for the T cell-inflamed tumor phenotype [22]. Clinical response to anti-PD-1 monoclonal antibody was reported to occur almost exclusively in patients with pre-existing T cell infiltrates in the region of PD-L1 upregulation [7, 23]. Following anti-PD-1 administration, these CD8^+^ T cells seemed to proliferate and expand to penetrate throughout the tumor, which correlated with tumor regression [7]. Altogether, our findings implicate that anti-PD-1/PD-L1 therapy may improve the unfavorable survival of the subset of endometrial cancers with TICs expressing high PD-L1.

Moreover, in the analysis of the associations between the TME protein expressions and TFI after primary adjuvant chemotherapy, high PD-L1 expression in TCs indicated a longer TFI (Fig. 3a), suggesting that prognostic impact of PD-L1 expression may be mediated by affected chemosensitivity, as TFI reportedly correlates with response to chemotherapy for recurrence and/or survival after recurrence in endometrial cancer [24–26]. This hypothesis may be supported by the published findings where upregulation of the PD-1/PD-L1 axis confers chemoresistance in some types of tumor [27–29]. Accordingly, our findings further suggest that anti-PD-1/PD-L1 therapy may attenuate chemoresistance in the patients with TICs expressing high PD-L1.

In the univariate and multivariate analyses of prognostic factors, besides high PD-L1 expression in TCs, high density of CD4^+^ TICs was found to be significant and independent for favorable OS (Table 5), being consistent with previous publications where high infiltration of CD4^+^ TILs was reported to be a favorable prognostic factor for some types of malignancy [30–32]. Besides, high density of CD4^+^ TICs was found to be associated with longer TFI (Fig. 3a), suggesting that helper T cells also may affect prognosis through involving chemosensitivity. The proliferation and differentiation into regulatory T cells of CD4^+^ T cells is reported to be manipulated by retinoic acid [33], STAT3 silencing [34], and DNGR-1 targeting [35], raising their therapeutic possibility. Further basic and clinical studies are warranted to verify our proposal.

The KEYNOTE-028 phase I study evaluated the safety and efficacy of pembrolizumab, an anti-PD-1 monoclonal antibody, in patients with PD-L1-positive advanced solid tumors [36]. Pembrolizumab demonstrated a favorable safety profile and durable antitumor activity in a subgroup of patients with heavily pretreated advanced PD-L1-positive endometrial cancer [36]. Currently, many phase II/III clinical trials of anti-PD-1/PD-L1 therapy in endometrial cancers are ongoing. Our above findings indicate that anti-PD1/PD-L1 therapies combined with conventional chemotherapeutics may be beneficial for the patients with poor prognosis due to high PD-L1 expression in TICs through improving chemosensitivity.

There exist only few reports on prognostic significances of the TME proteins in endometrial cancer so far. Regarding PD-L1 expression and survival, Kim et al. have recently reported on 183 primary endometrial cancers that high PD-L1 expression on immune cells was an independent prognostic factor for poor PFS [37]. Ikeda et al. have also reported on 32 endometrioid endometrial cancers that the cases with high PD-L1 mRNA expression in cancer tissues showed significantly longer PFS [38]. Yamashita et al. have recently reported on 149 endometrioid endometrial cancers that high PD-L1 expression in tumor cells was significantly associated with better PFS [39]. These findings are in line with our results that high PD-L1 expression in TCs was associated with better OS (Fig. 2a), while high PD-L1 on TICs was associated with worse OS (Fig. 2b). As for CD8 expression and survival, Yamashita et al. have reported that CD8^+^ TILs was significantly associated with better PFS [39]. Ikeda et al. also reported that high CD8 mRNA expression in tumor tissues was significantly associated with longer PFS [38]. These findings are consistent with our result that high density of CD8^+^ TICs correlated with better OS (Fig. 2f). Bellone et al. have recently reported on 131 endometrial cancers that POLE-mutated tumors were associated with improved PFS and displayed increased numbers of CD4^+^ and CD8^+^ TILs as compared to wild-type POLE tumors, and that PD-1 was overexpressed in TILs from POLE-mutated vs. wild-type-tumors [40]. In our study, MSI was associated with high PD-L1 expression in TICs (Fig. 3b), which was significantly associated with worse OS (Fig. 2b). POLE-mutated endometrial cancers have been reported to be MSS in a couple of studies including this article [40–42]. Therefore, it is plausible that POLE-mutated tumors and MSI tumors may have the opposite prognostic features. As regards the relationship between PD-L1 expression and clinicopathologic features, Mo et al. reported on 75 endometrial cancers that PD-L1 expression in TICs was more frequently found in the moderately and poorly-differentiated tumors and type II than in the type I tumors [43], being in line with our finding that high PD-L1 expression in TICs was associated with non-endometrioid histology and non-G1 (Table 4). Further studies are warranted to clarify the clinical and prognostic significance of the TME status in endometrial cancer.

The present study still contains some limitations. The retrospective study design potentially causes selection biases. The number of studied samples is relatively small. The evaluation method for the TME protein expression is mainly based on semi-quantitative analyses. Nevertheless, the treatment strategy was almost consistent throughout the study period, and most importantly the follow-up duration was much longer than the former studies (median, 132 vs. 30.3–38 months [37, 39]), supporting the validity of our survival data.

## Conclusions

We have demonstrated here that high PD-L1 in TCs was associated with better OS, while high PD-L1 in TICs was associated with worse OS. High PD-L1 in TICs exhibited associations with high densities of CD8^+^ TILs and CD68^+^ TAMs, and MSI, while high PD-L1 in TCs correlated with longer TFI. High density of CD4^+^ TICs correlated with better OS and longer TFI. Univariate and multivariate analyses exhibited that high PD-L1 in TCs and high density of CD4^+^ TICs were significant and independent prognostic factors for favorable OS. The current findings indicate that PD-L1 and CD4^+^ helper T cells may be reasonable targets for improving survival via enhancing chemosensitivity, providing useful information for combining immunotherapies into the therapeutic strategy for endometrial carcinoma.

## Data Availability

The datasets used and/or analyzed during the current study are available from the corresponding author on reasonable request.

## Abbreviations

FIGO: International Federation of Gynecology and Obstetrics
IHC: Immunohistochemistry
LVI: Lymphovascular space invasion
MSI: Microsatellite instability
MSS: Microsatellite stable
OS: Overall survival
PD-L1: Programmed cell death-ligand 1
PD-1: Programmed cell death-1
PFS: Progression-free survival
TAM: Tumor-associated macrophage
TCs: Tumor cells
TFI: Treatment-free interval
TICs: Tumor-infiltrating immune cells
TILs: Tumor infiltrating lymphocytes
TME: Tumor microenvironment

## References

[1] GLOBOCAN 2018 [Available from: http://gco.iarc.fr/].

[2] Siegel RL, Miller KD, Jemal A (2019). Cancer statistics, 2019. CA Cancer J Clin.

[3] Salvesen HB, Haldorsen IS, Trovik J (2012). Markers for individualised therapy in endometrial carcinoma. Lancet Oncol.

[4] Moxley KM, McMeekin DS (2010). Endometrial carcinoma: a review of chemotherapy, drug resistance, and the search for new agents. Oncologist.

[5] Okazaki T, Honjo T (2007). PD-1 and PD-1 ligands: from discovery to clinical application. Int Immunol.

[6] Li X, Shao C, Shi Y, Han W (2018). Lessons learned from the blockade of immune checkpoints in cancer immunotherapy. J Hematol Oncol.

[7] Tumeh PC, Harview CL, Yearley JH, Shintaku IP, Taylor EJ, Robert L, Chmielowski B, Spasic M, Henry G, Ciobanu V (2014). PD-1 blockade induces responses by inhibiting adaptive immune resistance. Nature.

[8] Teng MW, Ngiow SF, Ribas A, Smyth MJ (2015). Classifying cancers based on T-cell infiltration and PD-L1. Cancer Res.

[9] Webb JR, Milne K, Kroeger DR, Nelson BH (2016). PD-L1 expression is associated with tumor-infiltrating T cells and favorable prognosis in high-grade serous ovarian cancer. Gynecol Oncol.

[10] Darb-Esfahani S, Kunze CA, Kulbe H, Sehouli J, Wienert S, Lindner J, Budczies J, Bockmayr M, Dietel M, Denkert C (2016). Prognostic impact of programmed cell death-1 (PD-1) and PD-ligand 1 (PD-L1) expression in cancer cells and tumor-infiltrating lymphocytes in ovarian high grade serous carcinoma. Oncotarget.

[11] Hamanishi J, Mandai M, Iwasaki M, Okazaki T, Tanaka Y, Yamaguchi K, Higuchi T, Yagi H, Takakura K, Minato N (2007). Programmed cell death 1 ligand 1 and tumor-infiltrating CD8+ T lymphocytes are prognostic factors of human ovarian cancer. Proc Natl Acad Sci U S A.

[12] Le DT, Uram JN, Wang H, Bartlett BR, Kemberling H, Eyring AD, Skora AD, Luber BS, Azad NS, Laheru D (2015). PD-1 blockade in tumors with mismatch-repair deficiency. N Engl J Med.

[13] Dudley JC, Lin MT, Le DT, Eshleman JR (2016). Microsatellite instability as a biomarker for PD-1 blockade. Clin Cancer Res.

[14] Di Tucci C, Capone C, Galati G, Iacobelli V, Schiavi MC, Di Donato V, Muzii L, Panici PB (2019). Immunotherapy in endometrial cancer: new scenarios on the horizon. J Gynecol Oncol.

[15] Abe A, Minaguchi T, Ochi H, Onuki M, Okada S, Matsumoto K, Satoh T, Oki A, Yoshikawa H (2013). PIK3CA overexpression is a possible prognostic factor for favorable survival in ovarian clear cell carcinoma. Hum Pathol.

[16] Boland CR, Thibodeau SN, Hamilton SR, Sidransky D, Eshleman JR, Burt RW, Meltzer SJ, Rodriguez-Bigas MA, Fodde R, Ranzani GN. A National Cancer Institute workshop on microsatellite instability for cancer detection and familial predisposition: development of international criteria for the determination of microsatellite instability in colorectal cancer. In: AACR. 1998. 9823339

[17] Eo S-H, Hong S-M, Cho H. K-adaptive partitioning for survival data: the Kaps add-on package for R. arXiv preprint arXiv. 2013;13064615.

[18] Yang Y, Li CW, Chan LC, Wei Y, Hsu JM, Xia W, Cha JH, Hou J, Hsu JL, Sun L (2018). Exosomal PD-L1 harbors active defense function to suppress T cell killing of breast cancer cells and promote tumor growth. Cell Res.

[19] Theodoraki MN, Yerneni SS, Hoffmann TK, Gooding WE, Whiteside TL (2018). Clinical significance of PD-L1(+) Exosomes in plasma of head and neck Cancer patients. Clin Cancer Res.

[20] Okuma Y, Hosomi Y, Nakahara Y, Watanabe K, Sagawa Y, Homma S (2017). High plasma levels of soluble programmed cell death ligand 1 are prognostic for reduced survival in advanced lung cancer. Lung Cancer.

[21] Okuma Y, Wakui H, Utsumi H, Sagawa Y, Hosomi Y, Kuwano K, Homma S (2018). Soluble programmed cell death ligand 1 as a novel biomarker for Nivolumab therapy for non-small-cell lung Cancer. Clin Lung Cancer.

[22] Gajewski TF (2015). The next hurdle in Cancer immunotherapy: overcoming the non-T-cell-inflamed tumor microenvironment. Semin Oncol.

[23] Topalian SL, Hodi FS, Brahmer JR, Gettinger SN, Smith DC, McDermott DF, Powderly JD, Carvajal RD, Sosman JA, Atkins MB (2012). Safety, activity, and immune correlates of anti-PD-1 antibody in cancer. N Engl J Med.

[24] Shimamoto K, Saito T, Okadome M, Shimokawa M (2014). Prognostic significance of the treatment-free interval in patients with recurrent endometrial cancer. Eur J Obstet Gynecol Reprod Biol.

[25] Miyake T, Ueda Y, Egawa-Takata T, Matsuzaki S, Yokoyama T, Miyoshi Y, Kimura T, Yoshino K, Fujita M, Yamasaki M (2011). Recurrent endometrial carcinoma: prognosis for patients with recurrence within 6 to 12 months is worse relative to those relapsing at 12 months or later. Am J Obstet Gynecol.

[26] Moore KN, Tian C, McMeekin DS, Thigpen JT, Randall ME, Gallion HH (2010). Does the progression-free interval after primary chemotherapy predict survival after salvage chemotherapy in advanced and recurrent endometrial cancer? A gynecologic oncology group ancillary data analysis. Cancer.

[27] Black M, Barsoum IB, Truesdell P, Cotechini T, Macdonald-Goodfellow SK, Petroff M, Siemens DR, Koti M, Craig AW, Graham CH (2016). Activation of the PD-1/PD-L1 immune checkpoint confers tumor cell chemoresistance associated with increased metastasis. Oncotarget.

[28] Xu S, Tao Z, Hai B, Liang H, Shi Y, Wang T, Song W, Chen Y, OuYang J, Chen J (2016). miR-424(322) reverses chemoresistance via T-cell immune response activation by blocking the PD-L1 immune checkpoint. Nat Commun.

[29] Zhang P, Ma Y, Lv C, Huang M, Li M, Dong B, Liu X, An G, Zhang W, Zhang J (2016). Upregulation of programmed cell death ligand 1 promotes resistance response in non-small-cell lung cancer patients treated with neo-adjuvant chemotherapy. Cancer Sci.

[30] Chen K, Zhu Z, Zhang N, Cheng G, Zhang F, Jin J, Wu J, Ying L, Mao W, Su D (2017). Tumor-infiltrating CD4+ lymphocytes predict a favorable survival in patients with operable esophageal squamous cell carcinoma. Med Sci Monit.

[31] Nguyen N, Bellile E, Thomas D, McHugh J, Rozek L, Virani S, Peterson L, Carey TE, Walline H, Moyer J (2016). Tumor infiltrating lymphocytes and survival in patients with head and neck squamous cell carcinoma. Head Neck.

[32] Nejati R, Goldstein JB, Halperin DM, Wang H, Hejazi N, Rashid A, Katz MH, Lee JE, Fleming JB, Rodriguez-Canales J (2017). Prognostic significance of tumor-infiltrating lymphocytes in patients with pancreatic ductal adenocarcinoma treated with Neoadjuvant chemotherapy. Pancreas.

[33] Brown CC, Noelle RJ (2015). Seeing through the dark: new insights into the immune regulatory functions of vitamin a. Eur J Immunol.

[34] Sanseverino I, Purificato C, Varano B, Conti L, Gessani S, Gauzzi MC (2014). STAT3-silenced human dendritic cells have an enhanced ability to prime IFNgamma production by both alphabeta and gammadelta T lymphocytes. Immunobiology.

[35] Joffre OP, Sancho D, Zelenay S, Keller AM, Reis e Sousa C (2010). Efficient and versatile manipulation of the peripheral CD4+ T-cell compartment by antigen targeting to DNGR-1/CLEC9A. Eur J Immunol.

[36] Ott PA, Bang YJ, Berton-Rigaud D, Elez E, Pishvaian MJ, Rugo HS, Puzanov I, Mehnert JM, Aung KL, Lopez J (2017). Safety and antitumor activity of Pembrolizumab in advanced programmed death ligand 1-positive endometrial Cancer: results from the KEYNOTE-028 study. J Clin Oncol.

[37] Kim J, Kim S, Lee HS, Yang W, Cho H, Chay DB, Cho SJ, Hong S, Kim JH (2018). Prognostic implication of programmed cell death 1 protein and its ligand expressions in endometrial cancer. Gynecol Oncol.

[38] Ikeda Y, Kiyotani K, Yew PY, Sato S, Imai Y, Yamaguchi R, Miyano S, Fujiwara K, Hasegawa K, Nakamura Y (2017). Clinical significance of T cell clonality and expression levels of immune-related genes in endometrial cancer. Oncol Rep.

[39] Yamashita H, Nakayama K, Ishikawa M, Nakamura K, Ishibashi T, Sanuki K, Ono R, Sasamori H, Minamoto T, Iida K (2018). Microsatellite instability is a biomarker for immune checkpoint inhibitors in endometrial cancer. Oncotarget.

[40] Bellone S, Bignotti E, Lonardi S, Ferrari F, Centritto F, Masserdotti A, Pettinella F, Black J, Menderes G, Altwerger G (2017). Polymerase epsilon (POLE) ultra-mutation in uterine tumors correlates with T lymphocyte infiltration and increased resistance to platinum-based chemotherapy in vitro. Gynecol Oncol.

[41] Konstantinopoulos PA, Matulonis UA (2015). POLE mutations as an alternative pathway for microsatellite instability in endometrial cancer: implications for lynch syndrome testing. Cancer.

[42] Billingsley CC, Cohn DE, Mutch DG, Stephens JA, Suarez AA, Goodfellow PJ (2015). Polymerase varepsilon (POLE) mutations in endometrial cancer: clinical outcomes and implications for lynch syndrome testing. Cancer.

[43] Mo Z, Liu J, Zhang Q, Chen Z, Mei J, Liu L, Yang S, Li H, Zhou L, You Z (2016). Expression of PD-1, PD-L1 and PD-L2 is associated with differentiation status and histological type of endometrial cancer. Oncol Lett.

